# A Case Study on Post-traumatic Stress Disorder, Prolonged Grief Disorder, and Adjustment Disorder

**DOI:** 10.7759/cureus.80595

**Published:** 2025-03-14

**Authors:** Gunjan Y Trivedi, Parishi Thakore

**Affiliations:** 1 Society for Energy and Emotions, Wellness Space, Ahmedabad, IND

**Keywords:** icd-11 disorders specifically associated with stress, inner child integration therapy, post-traumatic stress disorder, prolonged grief disorder, reconsolidation of traumatic memories

## Abstract

In the International Classification of Disease (ICD)-11, the World Health Organization (WHO) has made significant changes to simplify the diagnosis, especially in disorders specifically associated with stress. These changes help differentiate between post-traumatic stress disorder (PTSD), prolonged grief disorder (PGD), and adjustment disorder (AjD). This case study covers an individual's journey with signs of all three disorders.

After suddenly losing his role in a family-owned business and the unexpected death of his son in his 20s, a man in his early 50s experienced significant mental anguish. Because of these events, he developed severe depression, insomnia, unrelenting grief, and flashbacks linked to the traumatic event (loss of the child). A psychiatric evaluation showed that the symptoms required medication. The depression, anxiety, sleep quality, and well-being assessments showed severe distress. The PTSD and PGD assessment, assisted by the therapist, highlighted the presence of symptoms related to both.

Reconsolidation of traumatic memories (RTM) and inner child integration therapy were used to address the PTSD symptoms at first. Over six months, the levels of depression, sleep disruption, and anxiety went down significantly. The individual was able to return to normal functioning. Medication was phased out with the support and collaboration of the psychiatrist. The patient eventually came to terms with the events and started working on new projects, which confirmed the progress validated by repeated assessments of PTSD, depression, and insomnia.

This case shows the utility of ICD-11's updated diagnostic criteria for identifying the difference between stress-related disorders. Correct assessment is key to understanding and addressing the core issue.

## Introduction

The World Health Organization (WHO), in the International Classification of Disease (ICD)-11, has made a distinction between mental disorders specifically associated with stress [1]. After nearly three decades of ICD-10, WHO has focused on improving the clinical utility and applicability of the diagnoses, one of the key factors driving the new diagnoses. Clinicians and mental health practitioners need to map the Diagnostic and Statistical Manual (DSM)-5 and ICD-10 assessments to the new ICD-11-based assessment in a way that provides distinct assessment.

This single-case study explores the therapeutic intervention of an individual who had symptoms from several stress-related disorders highlighted in ICD-11, specifically post-traumatic stress disorder (6B40: PTSD), prolonged grief disorder (6B42: PGD), and adjustment disorder (6B43:AjD) [2]. ICD-11 has also made an obvious distinction between complex post-traumatic stress disorder (CPTSD) and PTSD, still not called out in DSM-5. Recent evidence has highlighted that individuals with PGD are also likely to present with symptoms of PTSD, depressive symptoms, and anxiety [3]. Each of the assessments is summarized below based on the ICD-11 perspective.

PGD is linked to grieving, a natural response to loss, but for some individuals, the grieving process becomes prolonged and debilitating, leading to a condition known as PGD. Pre-existing depression, sudden loss, and death of a child are some of the risk factors for PGD. Recognized in both the DSM-5-TR (Text Revision) and ICD-11, PGD is characterized by persistent, pervasive grief that extends beyond the culturally or societally expected mourning periods. This disorder can significantly impair an individual's emotional, social, and occupational functioning, underscoring the importance of timely identification and intervention.

The PTSD diagnosis is proposed to consist of a reduced set of six symptoms, organized into three core elements, each of which is required for the diagnosis: re-experiencing the traumatic event(s) in the present, accompanied by feelings of fear or horror; avoidance of traumatic reminders; and a sense of current threat, manifested by excessive hypervigilance or an enhanced startle reaction. The syndrome has fear or horror at its heart with a focus on the re-experiencing of the trauma memory and consequent avoidance and hypervigilance. In contrast, CPTSD extends beyond these core PTSD symptoms and includes disturbances in self-organization (DSO), which manifest as affective dysregulation, negative self-concept, and interpersonal difficulties [4].

AjD, as defined in the ICD-11, is a condition triggered by identifiable stressors, such as significant life changes or adverse events, that result in marked distress and difficulties in functioning. Unlike typical stress reactions, the symptoms of AjD are disproportionate to the intensity of the stressor and persist for longer than expected in the given cultural context. Core features include preoccupation with the stressor, difficulty adapting to the change, and associated emotional disturbances such as anxiety, sadness, or behavioral symptoms. Notably, the symptoms arise within one month of the stressor and typically resolve within six months unless the stressor persists.

## Case presentation

The individual, a man in his early 50s, sought therapy following two significant life stressors: the "sudden" loss of his professional role within a family-owned business and the tragic death of his adult son. Unfortunately, the business triggers occurred a few months before the loss of the son, and therefore the individual was already emotionally disturbed when the son passed away. The series of events led to extreme emotional distress, high levels of depression, sleep disturbances, and flashbacks of the event (loss of the adult son). After the incidents, there was initial denial, and eventually, about a year later, the individual sought psychiatric help. About two years after seeking psychiatric help, the individual reached out to us. During the first visit, he was on two psychiatric medications, selective serotonin reuptake inhibitor (SSRI) and serotonin and norepinephrine reuptake inhibitor (SNRI) prescribed by the psychiatrist. SSRIs are a class of drugs used to treat depression and other mental health conditions, and SNRIs are a class of medications used to treat depression, anxiety, and chronic pain. Presenting symptoms included persistent flashbacks and avoidance related to the son's death, linked to a persistent feeling of sadness, loss of interest in activities such as physical activities or social connections, and a feeling of hopelessness. The individual had low energy, found it difficult to focus, and experienced disrupted sleep despite the medications.

The individual grew up in a close-knit family and lost his father before the age of 10. The family business served for decades, providing financial security and a sense of purpose. However, the individual's professional role in the business ended abruptly due to interpersonal conflicts, leaving him feeling abandoned and undervalued. A few months later, the individual lost his young son due to a sudden heart issue. This aggravated his emotional pain, with increased feelings of helplessness and despair. Both losses deeply affected his sense of self-worth and ability to cope with daily life functions. Table 1 highlights presenting symptoms in the context of PGD and PTSD.

**Table 1:** Presenting symptoms and overlap between PGD and PTSD PGD, prolonged grief disorder; PTSD, post-traumatic stress disorder

Presenting symptoms	PGD	PTSD
Persistent grief and sadness over the loss of his son	x	x
Flashbacks of the event of the loss (specific situations)		x
Avoiding thoughts, conversations, or places that bring back memories of his son’s loss		x
Feelings of betrayal and resentment toward the business partner		
Extreme rage and displaced anger outbursts	x	
Adjusting to the new family dynamics after the loss of an adult child and separation from a business partner		
Insomnia, depression, and anxiety	x	x
Struggles with self-worth and identity stemming from both personal and professional losses	x	
Overwhelming emotions of grief and sadness related to the death of his son affected daily functioning and relationships		

The individual developed severe insomnia, high levels of depression, and anxiety. He was prescribed the medications, which he used for two years with limited effectiveness. After the son’s death, his emotional health declined further, marked by profound grief, guilt, and difficulty in processing his feelings. 

The individual's initial self-assessment indicated severe depression, with a Major Depression Inventory (MDI) score of 26, and moderate to severe anxiety, as reflected by a generalized anxiety disorder (GAD)-7 score of 10 [5,6]. He experienced significant sleep disturbances with an Insomnia Severity Index (ISI) score of 19, persistent sadness, and grief related to the loss of his child resulting in poor well-being [7,8]. Additionally, he struggled with resentment and feelings over professional setbacks. The symptoms of insomnia further impacted his daily functioning.

Self-assessment assisted by the therapist for PGD (using the Aarhus PGD scale for ICD-11) and PTSD (using the International Trauma Questionnaire (ITQ)) indicated several PGD symptoms were present, and PTSD assessment was positive confirming the presence of core symptoms of PTSD [9,10]. Given the strong presence of re-experiencing, avoidance, and a sense of threat as presenting symptoms, further work was carried out with PTSD as the core focus of the assessment. Consequently, the index trauma event(s) were identified for intervention in PTSD. Frequent flashbacks, a sense of threat, and avoidance related to the index trauma events were observed. It is important to note that the three index trauma events included two specific events related to the loss of a son and one event involving a business partner, which occurred much earlier than the loss of the professional role. Suicide risk was assessed using the Ask Suicide Screening Questions Toolkit (ASQ), which showed no acute suicide risk [11].

Initial sessions mainly focused on PTSD, specifically index trauma events using reconsolidation of traumatic memories (RTM) and inner child integration therapies. RTM is an emerging approach and a possible alternative to current interventions such as CBT (cognitive behavioral therapy) or EMDR (eye movement desensitization reprocessing) for addressing trauma [12]. RTM is non-traumatizing and does not require the client to “confront” their traumatic memories due to its unique design [13]. RTM uses double dissociation to avoid direct traumatization, which was particularly relevant for the case given the resistance to talk or go into event details. A psychotherapy approach involves four steps: (a) safe space anchoring, (b) emotional bridges to regress to specific events, (c) processing the events, and, finally, (d) integration and future pacing [14]. Finally, the events related to AjDs were also worked upon.

There was resistance to discussing the details of the trauma event, which is usual when there is a very high SUDS (subjective units of distress) level concerning the events. This is where the RTM intervention with double dissociation proved impactful. Depression level, after five sessions, went down when the individual was able to associate with the event and discuss it, with sadness. This was followed with a repeat PTSD assessment where all the core systems went down indicating negative PTSD. Table 2 provides details of specific assessments. Ajd was not conducted due to the overwhelming presentation of both PGD and PTSD, as confirmed by index trauma assessment [15,16]. Consistent with existing literature, a trauma-informed care approach was taken with a focus on improving sleep quality. Existing evidence supports sleep-focused treatment for bereavement and PGD where self-hypnosis, physical activity, and simple guidelines were provided [17,18].

**Table 2:** Assessments of WHO-5, MDI, GAD-7, and ISI before and after therapeutic intervention WHO-5: World Health Organization-five well-being index; GAD-7: generalized anxiety disorder-7; MDI: major depression inventory; ISI: insomnia severity index

Timing	Well-being (WHO-5), desired >52	Anxiety (GAD-7), desired >9	Depression (MDI), desired >19	Insomnia (ISI), desired >9
Before consultation	28	10	26	19
After 2 months	64	12	17	4
After 5 months	72	7	10	4

Table 2 and Table 3 highlight the progression in various scores improvement across multiple areas. His depression scores (MDI), insomnia, and anxiety levels returned to healthy levels. The improvement in depression and insomnia happened only after the successful RTM intervention on all three index trauma events. After that, the individual, with complete willingness, continued the consultations with a psychiatrist. The psychiatrist, upon confirmation of the progress with the individual and the therapist, eventually phased out the medications. The individual eventually reported acceptance regarding his son's death and initiated a new business venture. His sleep quality improved, and he started regular physical activity. 

**Table 3:** Trauma assessments before and after therapeutic interventions PTSD/DSO/CPTSD were assessed using the ITQ. PTSD, post-traumatic stress disorder; CPTSD, complex post-traumatic stress disorder; DSO: disturbance in self-organization; ITQ: International Trauma Questionnaire

Timing	Trauma assessment using ITQ	PTSD	DSO	CPTSD
Before therapy	PTSD	18	12	30
After 1 month of therapy	None	5	5	10
After 4 months of therapy	None	3	6	9

## Discussion

This case study supports the distinction covered in ICD-11 in identifying disorders specifically associated with stress, namely PTSD, PGD, and AjD. Given some overlap between the symptoms, applying an ICD-11-based definition to assess the core issue can help understand where to initiate the therapeutic intervention. The patient exhibited overlapping symptoms requiring careful clinical assessment (depression, anxiety, insomnia, PTSD, and PGD) and targeted treatments for each diagnostic category. AjD symptoms were present, but the assessment was not done, given the clear presence of the overlap between the PGD and PTSD symptoms. The assessment process and awareness of the uniqueness and differences between the symptoms are critical for improving the probability of success. For example, existing evidence highlights a high degree of co-occurrence between stress-related disorders; specifically, more than 70% of PGD cases also exhibit clinically significant levels of depression and PTSD symptoms [19].

The therapeutic intervention (RTM) is known to be effective in addressing PTSD symptoms and could help in CPTSD based on emerging evidence [12,13,20]. Given that PTSD was core to PGD, as highlighted by the index trauma events, the intervention initially focused on addressing PTSD, despite the resistance from the individual to work on it. This was followed by inner child integration therapy to work through the decisions that can help reduce depression, anxiety, and overthinking issues. 

The gradual decrease in psychiatric medication highlights the need to integrate psychotherapy and psychiatric drugs, especially when the symptoms are extreme. The collaboration between the therapist and the psychiatrist also highlights the effectiveness of a multidisciplinary treatment approach.

Although the outcome is favorable, the case study highlights the difficulties associated with navigating profound loss and trauma. The initial reluctance toward therapy, especially with emotionally charged traumatic experiences, underscores the importance of patience, trust development, and personalized therapeutic intervention supported by the correct ICD-11 assessment. Future studies should investigate the best sequence of trauma-focused treatment and bereavement therapies to improve therapeutic effectiveness.

## Conclusions

This case study highlights the negative impact of personal and professional losses in PTSD (based on the ICD-11 definition). Based on the ICD-11 assessment and the interview with the individual, the treatment revolved around the assessment of PTSD despite the presence of PGD. This is an important insight from the case for potential re-application, where the individual's inputs were considered along with the assessment. This led to the identification of index trauma events and the intervention (RTM). The case also highlights the importance of collaboration with the psychiatrist during the therapeutic journey. This integrative approach enabled the client to regain hope and purpose in the face of significant losses. Slow and steady progress, along with collaboration and the availability of multiple resources, further facilitated not only recovery but also the eventual phasing out of the medications.

The case study reinforces the importance of ICD-11’s new diagnostic framework in guiding clinicians toward accurate assessment, the key to planning effective intervention for disorders specifically associated with stress. The successful outcomes of the case highlight the need for a structured assessment, the importance of personal interviews, and the individual’s perspective, followed by an evidence-based approach focused on the core issues identified during the assessment.
